# Adenocarcinoma arising from an ectopic pancreas in the duodenum: a case report

**DOI:** 10.1186/s40792-019-0684-8

**Published:** 2019-08-06

**Authors:** Tsukasa Kaneko, Masanori Ohara, Kunishige Okamura, Aki Fujiwara-Kuroda, Daisuke Miyasaka, Takumi Yamabuki, Ryo Takahashi, Kazuteru Komuro, Masato Suzuoki, Nozomu Iwashiro, Mototsugu Kato, Noriko Kimura, Hiroshi Kijima, Toru Nakamura, Satoshi Hirano

**Affiliations:** 1 Department of Surgery, National Hospital Organization Hakodate National Hospital, Hakodate, Japan; 2 Department of Gastroenterology, National Hospital Organization Hakodate National Hospital, Hakodate, Japan; 3 Department of Pathology, National Hospital Organization Hakodate National Hospital, Hakodate, Japan; 4 Department of Surgery, Japanese Red Cross Hakodate Hospital, Hakodate, Japan; 5 Department of Pathology and Bioscience, Graduate School of Medicine, Hirosaki University, Hirosaki, Japan; 6 Department of Gastroenterological Surgery II, Faculty of Medicine, Hokkaido University, Sapporo, Japan

**Keywords:** Ectopic pancreas, Distal gastrectomy, Duodenal adenocarcinoma, Cancer-induced vomiting

## Abstract

**Background:**

The malignant transformation of an ectopic pancreas in the duodenum is extremely rare. Herein, we report a case of an adenocarcinoma that arose from an ectopic pancreas. We also reviewed 14 cases of malignant transformations arising from an ectopic pancreas in the duodenum that were previously published.

**Case presentation:**

An 81-year-old man with a 1-month history of vomiting was admitted to our institution. Esophagogastroduodenoscopy (EGD) and computed tomography (CT) scans revealed an obstruction at the first part of the duodenum. A distal gastrectomy was performed for diagnostic and therapeutic purposes. The histopathological examination of the resected specimen showed adenocarcinoma that arose from an ectopic pancreas (Heinrich type 1). The patient is alive without relapse at 18 months of follow-up.

**Conclusions:**

Adenocarcinoma that arises from an ectopic pancreas should be considered when an obstruction is identified in the duodenum.

## Background

An ectopic pancreas, often found during surgery or biopsy, is defined as an uncommon pancreatic tissue outside the normal pancreas, which lacks any connection to the normal pancreas, and has its own vascular and ductal systems [1]. The frequency of the occurrence of ectopic pancreatic tissue is found in 0.25% of abdominal surgeries and 1.2% of gastrectomy operations, and its frequency at autopsy has been reported to be 0.55–13.7% [2]. Ectopic pancreatic tissue has been found in both abdominal and extra-abdominal locations, but is mainly encountered in the duodenum (25–35%) [3] and stomach (25–60%) [4], though mesocolon [5, 6] and Meckel’s diverticulum [7] are also other rare sites. Malignant transformations that arise from ectopic pancreatic tissue are extremely rare, and there are only 14 reported cases in the literature. Here, we report a case of adenocarcinoma that arose from an ectopic pancreas in the first part of the duodenum.

## Case presentation

**Fig. 1:**
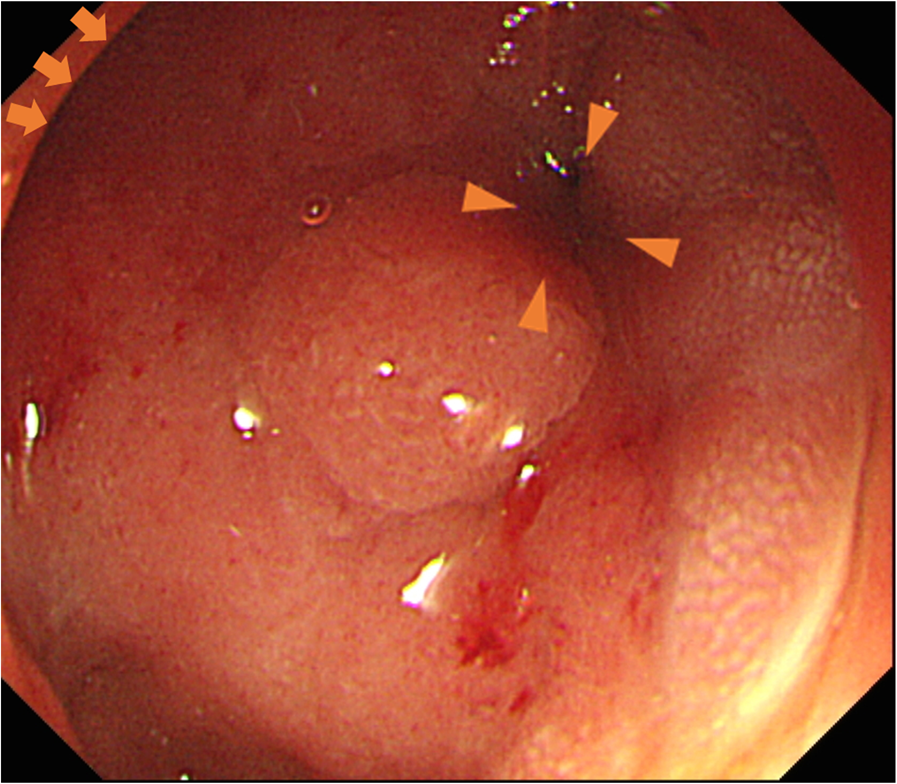
Esophagogastroduodenoscopy showing the obstruction at the first part of the duodenum. Arrow: pylorus ring. Arrow head: obstruction of the first part of the duodenum

**Fig. 2:**
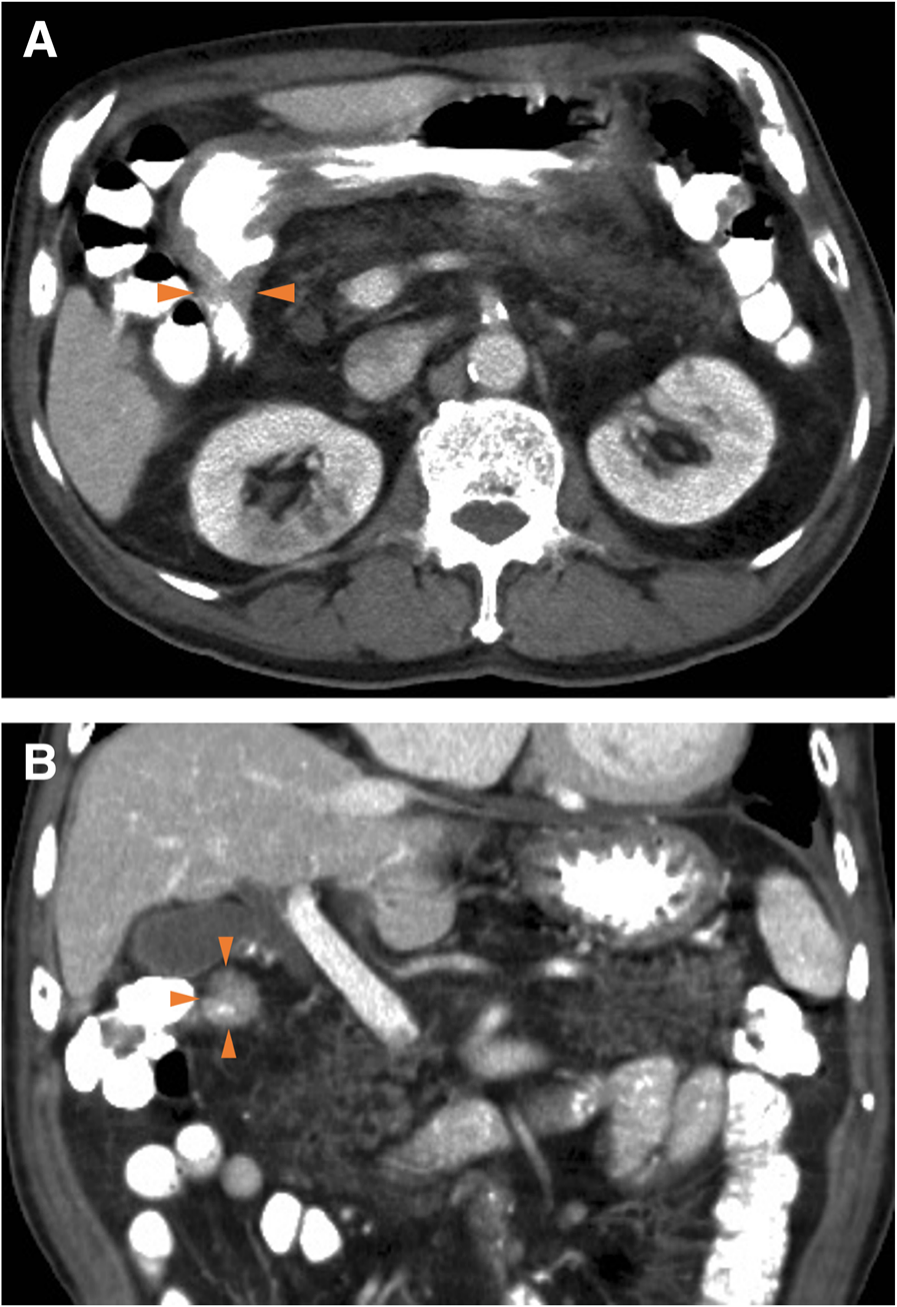
Enhanced multi-detector row computed tomography (MDCT) image showing the wall thickness in the duodenum in the transverse (**a**) and the coronal (**b**) planes. Arrow heads: wall thickness of the first part of the duodenum

An 81-year-old Japanese man was admitted to our institution with a 1-month history of vomiting. Although the patient did not complain of any obvious weight loss, he experienced daily persistent vomiting and always felt full. Past medical history was positive for chronic atrial fibrillation, chronic heart failure, Graves’ disease, hyperlipidemia, and benign prostatic hyperplasia. The patient had no previous surgical history. Serum tumor markers, such as carbohydrate antigen (CA) 19–9, CA 125, α-fetoprotein (AFP), and carcinoembryonic antigen (CEA), were all within normal ranges. An esophagogastroduodenoscopy (EGD) revealed a submucosal tumor-like lesion with a smooth surface involving the entire circumference of the first part of the duodenum. The demarcation line of the lesion was unclear (Fig. 1). We could not pass the endoscope beyond the first part of the duodenum because of duodenal stenosis. An endoscopic ultrasound (EUS) was not performed; enhanced multi-detector row computed tomography (enhanced MDCT) revealed increased wall thickness in the first part of the duodenum (Fig. 2). No swollen lymph nodes were detected. The forceps biopsy specimen from the submucosal tumor-like lesion did not show evidence of malignancy. As the possibility of a malignant tumor could not be ruled out clinically, a surgical resection was planned for diagnostic and therapeutic purposes.

**Fig. 3:**
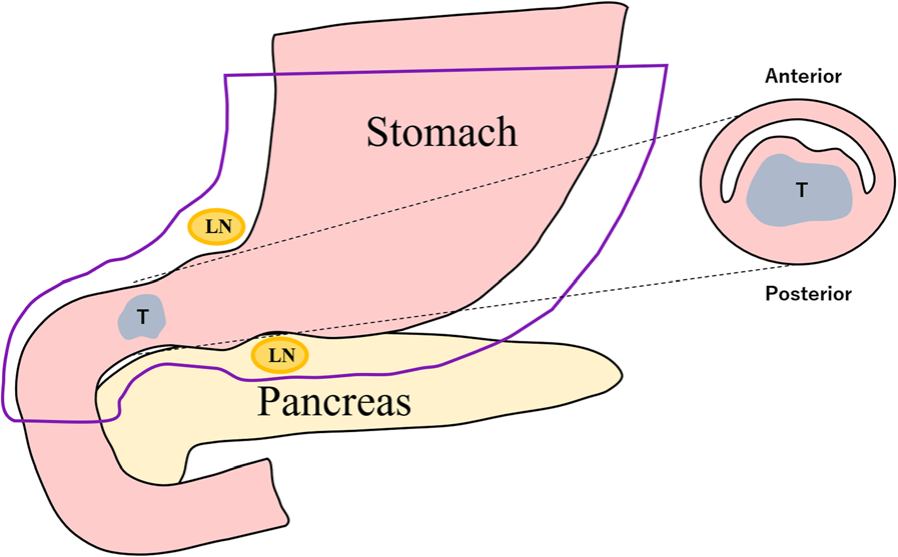
Schema of the operative findings. LN, Lymph node; T, Tumor

The patient subsequently underwent an open surgery. A hard mass was palpable in the duodenal bulb, which extended dorsally to the second part of the duodenum. After Kocherization of the duodenum, the area proximal to the pylorus ring to the end of the second part of the duodenum, where the tumor was not palpated, was resected (Fig. 3).

**Fig. 4:**
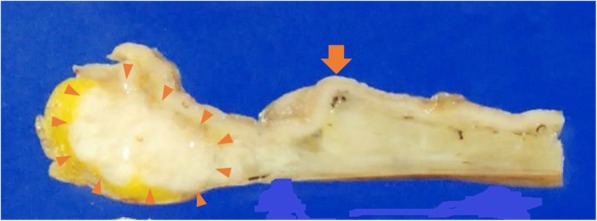
Resected specimen (gastric antrum and duodenum). Arrow heads: tumor. Arrow: pyloric ring

**Fig. 5:**
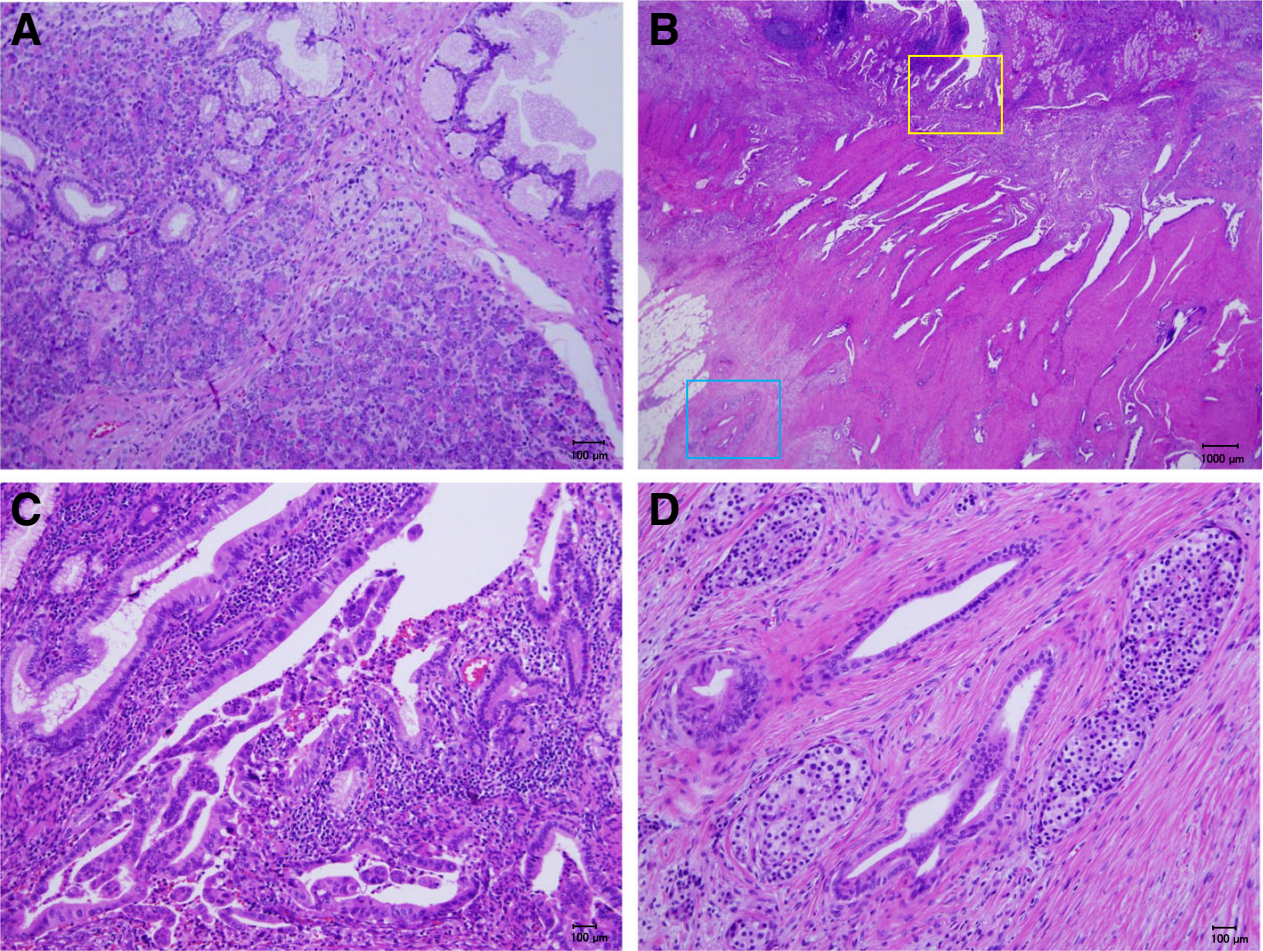
**a** Ectopic pancreas of the duodenum showing pancreatic ducts, acinar cells, and islets called Heinlich type1. **b** Adenocarcinoma in the duodenum mainly located in extra-mucosal layer, and combined with ectopic pancreas, which is consistent with carcinoma derived from ectopic pancreas. Magnified figures in the square are shown in **c** (yellow) and **d** (blue). **c** Papillary adenocarcinoma of the duodenum derived from ectopic pancreas. **d** Ectopic pancreatic tissue composed of pancreatic ducts and islets in duodenal muscle layer

A distal gastrectomy was performed. The tumor was 30 × 10 mm and located in the first part of the duodenum (Fig. 4). It was not continuous with the normal pancreas as revealed first by imaging and later confirmed during surgery. Microscopically, the tumor was diagnosed as a moderately differentiated adenocarcinoma that extended from the submucosal layer to the muscularis propria of the duodenum. Normal pancreatic tissue was observed adjacent to the tumor, suggesting the presence of an ectopic pancreas (Fig. 5). Surgical margins were negative for the presence of tumor cells. Moderate lymphatic invasion, moderate venous invasion, marked neural invasion, and metastases to both superior and inferior pyloric lymph nodes were observed. The adjacent ectopic pancreatic tissue had a microscopic appearance consistent with Heinrich’s type 1 [8, 9] and was characterized by the presence of ducts, islets, and acini. On immunohistochemical staining, the islets of the ectopic pancreas and the normal pancreas showed positive staining for chromogranin A, synaptophysin, neural cell adhesion molecule (NCAM), insulin, glucagon, and somatostatin. Based on these findings, our final diagnosis was of a ductal adenocarcinoma arising from an ectopic pancreas in the first part of the duodenum.

The patient was discharged 18 days after surgery, with no complications. Postoperative adjuvant chemotherapy was not administered. We performed a follow-up blood exam including tumor markers (CEA, CA 19–9) every 3 months and CT images every 6 months. The patient is alive without relapse, at 18 months of follow-up.

## Discussion

An ectopic pancreas is defined as an uncommon pancreatic tissue that exists outside the normal pancreas with no connection to it. The frequency of ectopic pancreatic tissue has been reported to be 0.25% in abdominal surgery [2], 25–35% of which was found in the duodenum [3].

Histopathologically, ectopic pancreatic tissue has been classified into four types by Heinrich [8, 9] depending on the presence or absence of pancreatic ducts, acini, and islet cells. The ectopic pancreas in our patient was located in the first part of the duodenum and contained ducts, acini, and islet cells, making it a Heinrich type 1 ectopic pancreas. The malignant transformation of ectopic pancreatic tissue is extremely rare, with a frequency that ranges from 0.7 to 1.8%, among all cases of ectopic pancreatic tissue [10, 11]. These tumors are usually located in the submucosal layer and only occasionally expand into the muscularis propria [1]. Jaervi and Lauren [12] have proposed three criteria for diagnosing carcinoma that arises from a heterotopic pancreas:

1. The tumor must be found within or close to the ectopic pancreatic tissue.
2. A direct transition must be observed between pancreatic structures and the carcinoma (malignant transformation of an ectopic pancreas must be differentiated from a metastatic deposit or a neoplastic invasion from a neighboring digestive cancer, especially from the stomach, the biliary tract, and the ectopic pancreas).
3. The non-neoplastic pancreatic tissue must comprise fully developed acini and ductal structures.

In this case, the adenocarcinoma was adjacent to the ectopic pancreas and located in the submucosal layer, away from the normal pancreas. No obvious cancer was seen in other organs. Therefore, we diagnosed the patient with adenocarcinoma of an ectopic pancreas in the duodenum.

To the best of our knowledge, 52 cases of malignant transformation arising from an ectopic pancreas, including the present case, have been reported in PubMed (keywords: ectopic OR heterotopic OR aberrant pancreas, carcinoma), 14 of which were malignant transformation arising from an ectopic pancreas in the duodenum (Table 1) [13–23]. The mean age of the patients in this group was 70.2 years (range 56–86 years), and eight patients were males and six were females. The mean tumor size was 27.9 mm (range 12–50 mm). Eleven of the 14 patients were pathologically diagnosed with adenocarcinoma (tubular adenocarcinoma, poorly differentiated adenocarcinoma, papillary adenocarcinoma, and mucinous adenocarcinoma), and about half of the carcinomas arose from Heinrich type 1 ectopic pancreatic tissue. In all except 2 of the 14 cases, the tumors were located in the first or second part of the duodenum.

**Table 1:** Review of case reports of adenocarcinoma arising from a heterotopic pancreas in the duodenum

Case	Year	Author	Age	Sex	Part of duodenum involved	Contrast effect on enhanced CT	Diagnostic approach	Operative method	Pathology	Outcome
1	1993	Tanaka	72	M	ND	ND	Operation	PD	Cancer	ND
2	1996	Inoue	81	F	ND	ND	Operation	DG	Adenocarcinoma + muc	ND
3	2006	Inoue	75	M	ND	ND	Operation	PPPD	Adenocarcinoma	ND
4	2007	Tison	72	M	Second portion, vater	ND	Operation	PD	Adenocarcinoma, CDHP	Death (16 months)
5	2007	Kawakami	65	F	Second portion, vater	Heterogeneously enhanced	Operation	SSPPD	Acinar cell carcinoma	Alive (19 months)
6	2008	Rosok	59	F	Proximal	Multi-cystic lesion	Operation	LR	IPMC	Alive (36 months)
7	2010	Inoue	75	M	Second portion	Homogeneously enhanced	Operation	PPPD	Adenocarcinoma	Alive (72 months)
8	2010	Bini	56	M	First portion	ND	Operation	PD	Adenocarcinoma	ND
9	2011	Stock	79	F	Fourth portion	ND	Operation	SD	Adenocarcinoma	ND
10	2012	Kinoshita	62	F	First portion	Heterogeneously enhanced	Operation	PD	Adenocarcinoma	Alive (12 months)
11	2013	Ginori	86	F	First portion	ND	Operation	STG + DR	Adenocarcinoma + muc	ND
12	2014	Endo	75	M	Second portion	ND	EUS-FNA	SSPPD	Adenocarcinoma	Alive (60 months)
13	2015	Fukino	62	M	Fourth portion	Poorly enhanced	Operation	SD	Adenocarcinoma	Death (33 months)
14	Present case		81	M	First portion	Same contrast effect as duodenum	Operation	DG	Adenocarcinoma	Alive (18 months)

*Abbreviations*: *M* male, *F* female, *ND* not described, *muc* mucinous carcinoma, *CDHP* cystic dystrophy in heterotopic pancreas, *IPMC* intraductal papillary-mucinous carcinoma, *EUS-FNA* endoscopic ultrasonography-guided fine-needle aspiration, *PD* pancreaticoduodenectomy, *DG* distal gastrectomy, *PPPD* pylorus-preserving pancreaticoduodenectomy, *SSPPD* subtotal stomach-preserving pancreaticoduodenectomy, *LR* laparoscopic resection of tumor and duodenal wall, *SD* segmental duodenectomy, *STG + DR* subtotal gastrectomy with duodenal bulb resection

Except for one patient, no other patient has reportedly been diagnosed with a malignant transformation arising from an ectopic pancreas prior to surgery. Endo et al. were able to diagnose an ectopic pancreas adenocarcinoma preoperatively using endoscopic ultrasonography-guided fine-needle aspiration (EUS-FNA) [22]. They suggested that EUS-FNA is a useful procedure for preoperative diagnosis in such cases [22] probably because ectopic pancreatic tissue is usually situated in the submucosal layer [1]. In our case, due to the obstruction of the duodenum, which made curative or palliative surgery necessary, EUS-FNA was not performed. Ordinary pancreatic cancer is characterized by an ischemic mass on enhanced CT; however, only one in six cases presented with low contrast effects. In the present case, the tumor was not distinctive on enhanced MDCT and the wall of the first part of the duodenum looked similar to the uninvolved parts.

Because few reports of malignant transformations arising from ectopic pancreatic tissue are available, no reports have compared prognosis between these patients and those with ordinary pancreatic cancer. An analysis of the eight cases of ectopic pancreatic tissue that had reported on patient prognoses after surgery, including the present case, revealed a 5-year survival rate of 64.3%. The corresponding survival rate for ordinary pancreatic cancer is about 10% [24]. In malignant transformations that arise from ectopic pancreatic tissue, gastrointestinal symptoms due to stenosis are easier to identify than in ordinary pancreatic cancer, which may result in a better prognosis in the former.

Some patients underwent postoperative adjuvant chemotherapy similar to patients with ordinary pancreatic cancer, such as S-1 or gemcitabine monotherapy [23]. However, in the present case, no postoperative adjuvant chemotherapy was performed, because no evidence of the efficacy of postoperative adjuvant chemotherapy for adenocarcinoma arising from ectopic pancreatic tissue in an old patient with low ADL exists.

## Conclusions

We reported an extremely rare case of an adenocarcinoma that arose from ectopic pancreatic tissue in the duodenum. Considering the rarity of this disease, gathering data from all cases of adenocarcinoma arising from ectopic pancreatic tissue in the duodenum will facilitate the development of diagnostic and treatment strategies.

## Data Availability

The dataset supporting the conclusions of this article is included within the article.

## Abbreviations

ADL: Activities of daily living
AFP: α-Fetoprotein
CA: Carbohydrate antigen
CEA: Carcinoembryonic antigen
CT: Computed tomography
EGD: Esophagogastroduodenoscopy
Enhanced MDCT: Enhanced multi-detector row computed tomography
EUS: Endoscopic ultrasound
EUS-FNA: Endoscopic ultrasonography-guided fine-needle aspiration
NCAM: Neural cell adhesion molecule
PD: Pancreaticoduodenectomy
